# Harnessing the power of AI in precision medicine: NGS-based therapeutic insights for colorectal cancer cohort

**DOI:** 10.3389/fonc.2024.1407465

**Published:** 2024-10-07

**Authors:** Victor Murcia Pienkowski, Piotr Skoczylas, Agata Zaremba, Stanisław Kłęk, Martyna Balawejder, Paweł Biernat, Weronika Czarnocka, Oskar Gniewek, Łukasz Grochowalski, Małgorzata Kamuda, Bartłomiej Król-Józaga, Joanna Marczyńska-Grzelak, Giovanni Mazzocco, Rafał Szatanek, Jakub Widawski, Joanna Welanyk, Zofia Orzeszko, Mirosław Szura, Grzegorz Torbicz, Maciej Borys, Łukasz Wohadlo, Michał Wysocki, Marek Karczewski, Beata Markowska, Tomasz Kucharczyk, Marek J. Piatek, Maciej Jasiński, Michał Warchoł, Jan Kaczmarczyk, Agnieszka Blum, Anna Sanecka-Duin

**Affiliations:** ^1^ AI Lab, Ardigen SA, Cracow, Poland; ^2^ Surgical Oncology Clinic, Maria Sklodowska-Curie National Research Institute of Oncology, Cracow, Poland; ^3^ Department of Surgery, Faculty of Health Sciences, Jagiellonian University Medical College, Cracow, Poland; ^4^ Department of General Surgery and Surgical Oncology, Ludwik Rydygier Memorial Hospital, Cracow, Poland; ^5^ Department of Oncological and General Surgery, Andrzej Frycz Modrzewski Krakow University, Cracow, Poland; ^6^ Department of General and Transplant Surgery, Poznan University of Medical Sciences, University Hospital, Poznan, Poland; ^7^ Holy Cross Cancer Center Clinic of Clinical Oncology, Kielce, Poland

**Keywords:** CRC, AI, precision medicine, synthetic lethality, neoantigens

## Abstract

**Purpose:**

Developing innovative precision and personalized cancer therapeutics is essential to enhance cancer survivability, particularly for prevalent cancer types such as colorectal cancer. This study aims to demonstrate various approaches for discovering new targets for precision therapies using artificial intelligence (AI) on a Polish cohort of colorectal cancer patients.

**Methods:**

We analyzed 71 patients with histopathologically confirmed advanced resectional colorectal adenocarcinoma. Whole exome sequencing was performed on tumor and peripheral blood samples, while RNA sequencing (RNAseq) was conducted on tumor samples. We employed three approaches to identify potential targets for personalized and precision therapies. First, using our in-house neoantigen calling pipeline, ARDentify, combined with an AI-based model trained on immunopeptidomics mass spectrometry data (ARDisplay), we identified neoepitopes in the cohort. Second, based on recurrent mutations found in our patient cohort, we selected corresponding cancer cell lines and utilized knock-out gene dependency scores to identify synthetic lethality genes. Third, an AI-based model trained on cancer cell line data was employed to identify cell lines with genomic profiles similar to selected patients. Copy number variants and recurrent single nucleotide variants in these cell lines, along with gene dependency data, were used to find personalized synthetic lethality pairs.

**Results:**

We identified approximately 8,700 unique neoepitopes, but none were shared by more than two patients, indicating limited potential for shared neoantigenic targets across our cohort. Additionally, we identified three synthetic lethality pairs: the well-known APC-CTNNB1 and BRAF-DUSP4 pairs, along with the recently described APC-TCF7L2 pair, which could be significant for patients with APC and BRAF variants. Furthermore, by leveraging the identification of similar cancer cell lines, we uncovered a potential gene pair, VPS4A and VPS4B, with therapeutic implications.

**Conclusion:**

Our study highlights three distinct approaches for identifying potential therapeutic targets in cancer patients. Each approach yielded valuable insights into our cohort, underscoring the relevance and utility of these methodologies in the development of precision and personalized cancer therapies. Importantly, we developed a novel AI model that aligns tumors with representative cell lines using RNAseq and methylation data. This model enables us to identify cell lines closely resembling patient tumors, facilitating accurate selection of models needed for in vitro validation.

## Introduction

Colorectal cancer (CRC) represents approximately 10% of all diagnosed cancer cases and is the second most frequent cause of cancer deaths worldwide (1, 2). Only in 2020, CRC caused 0.9 million deaths and was newly diagnosed in 1.9 million patients (3–5). The effectiveness of treating CRC, like with other cancers, hinges on implementing preventive strategies to inhibit metastasis and effectively manage metastatic disease (mCRC). Although the overall survival of patients with mCRC has improved over the last 20 years, mainly because of more effective surgical treatment of liver and lung metastases and the invention of anti-tumor drugs (6), mCRC, in most cases remains an incurable disease (7). The most commonly accepted origin of CRC proposes the transformation of a normal colon epithelium crypt into a benign adenomatous polyp, which then progresses into the actual disease (8). The standard treatment for CRC typically encompasses a multifaceted approach, integrating surgical intervention, chemotherapy, radiation therapy, targeted therapy, and immunotherapy. Tailoring the treatment plan to each patient’s needs hinges on several factors, including the cancer’s stage, tumor location, and the patient’s overall health. Traditionally, CRC staging and grading have served as fundamental pillars in classifying the disease, offering insights into its progression and aggressiveness. However, the recent integration of Consensus Molecular Subtypes (CMS) has enriched our understanding of CRC at a molecular level, providing invaluable insights for personalized treatment strategies, prognostic evaluation, and therapeutic decision-making in the management of colorectal cancer. The classification system of CRC, based on gene and transcriptome profiling, is divided into four CMS (9). According to this classification, CMS1 is characterized by hypermutation, microsatellite instability (MSI), and active response from the immune system. CMS2 is associated with high activation of the *Wnt* and *Myc* signal transduction pathways. CMS3, also known as the metabolic subtype, exhibits a high degree of chromosomal instability (CIN) and has relatively low somatic copy number alterations. CMS4 displays a mesenchymal phenotype with gene signatures engaged in angiogenesis, integrin binding and TGFβ signaling.

Personalized therapies involving autologous cancer-specific immune cells or cancer vaccines play a promising role in the treatment of CRC, particularly in the realm of immunotherapy. What is crucial for such therapies is the identification of tumor-specific antigens (TSAs), which are displayed at the tumor cell surface in the context of Human Leukocyte Antigen (HLA) molecules and are recognized by specific T cell receptors (TCRs) (10). TSAs differ from germline proteins and can be specifically recognized as non-self by the host immune system (11). Additionally, due to their foreign origin, they are not easily subjected to complex immune tolerance mechanisms (12). Genetic alterations that cause TSA generation include single-nucleotide variants (SNVs), insertions and deletions (indels), gene fusions, frameshift mutations, and structural variants (SVs) (11). These features of neoepitopes make them attractive candidates for novel cancer vaccines and adaptive-cell therapy (ACT) approaches utilizing TCRs.

Another promising approach that enables the discovery of cancer-specific therapy targets is the identification of synthetic lethality (SL) interactions. This phenomenon describes the condition where the loss or mutation of one gene alone is not lethal but in combination with the loss of another specific gene results in cell death or loss of viability (13). SL interactions are exploited in cancer treatment e.g. BRCA1/BRCA2 and PARP, where targeting an SL partner of a cancer-specific mutation can selectively eliminate cancer cells (13, 14). Identifying SL interactions in cancer cells typically requires laborious experimental validation, often leveraging CRISPR-Cas9 technology (15). However, the translation of experimental results obtained from cell lines into patients requires sophisticated comparative AI-based models as the heterogeneity of cancer in patients is extremely high (16). Our study introduces a novel approach that addresses several limitations in existing solutions such as Celligner (17). By integrating advanced Machine learning algorithms, incorporating batch corrections, and utilizing a comprehensive dataset including multiple types of omics data (RNAseq and methylation datasets), our approach offers significant improvements in applicability.

Recent studies have demonstrated the effectiveness of various Deep Learning (DL) approaches in enhancing predictive capabilities and integrating diverse data types (18). For instance, a generative model based on a conditional Variational AutoEncoder (VAE) enables the identification of new molecules with desired properties (19). Single-cell studies have utilized deep generative models to learn joint embeddings, integrating multiple data modalities and compensating for missing information (20). In the realm of microbiology, a multi-omics deep learning model accurately predicts genome-wide concentrations and growth dynamics of Escherichia coli (21). Additionally, Multimodal Variational AutoEncoder (MVAE) low-dimensional embeddings have proven superior in predicting drug-protein interactions (22). These advancements highlight the transformative potential of deep learning and multi-omics integration in various research applications.

**Figure 1:**
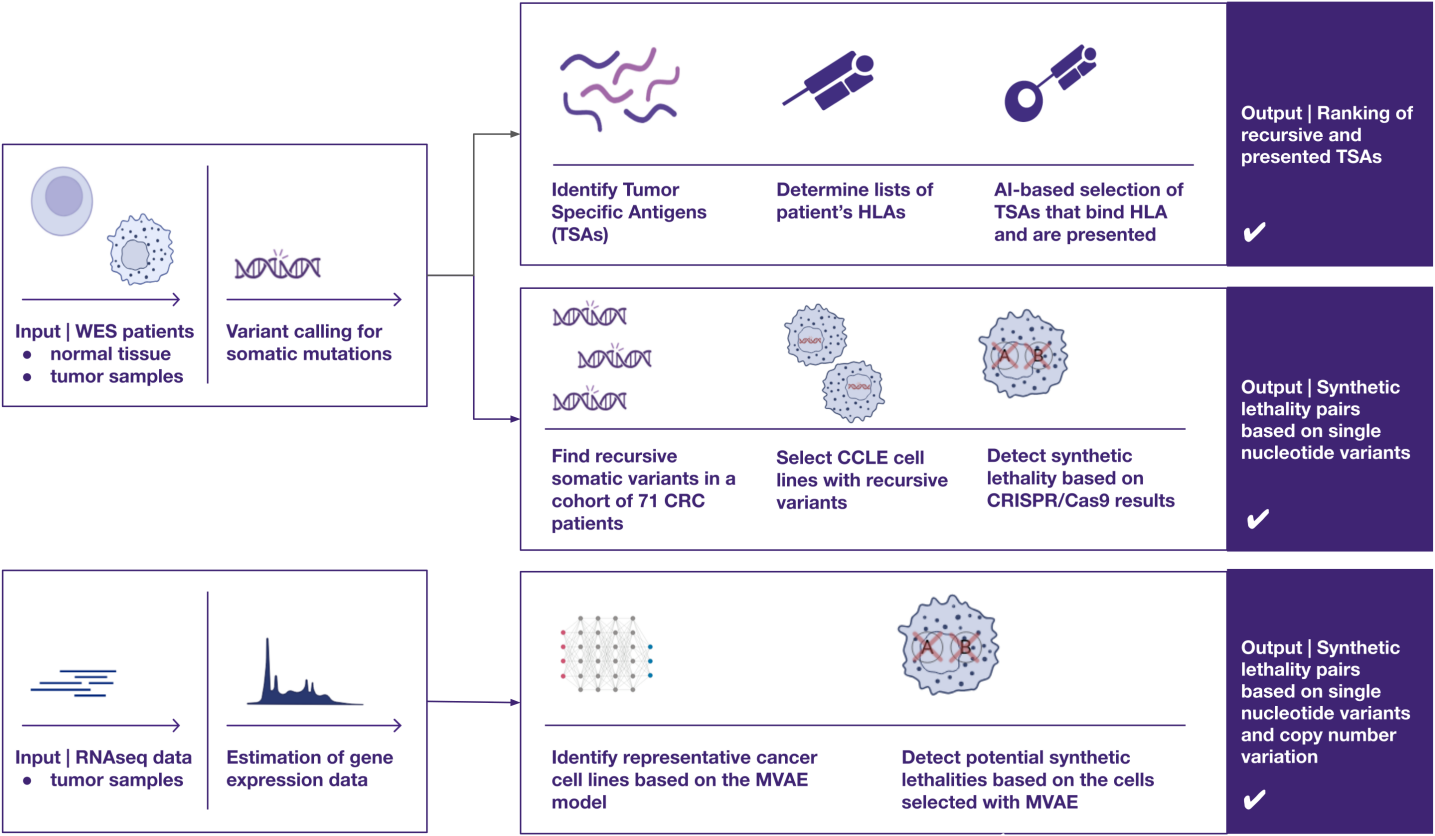
A visual overview of three distinct approaches for identifying therapeutic targets in cancer, utilizing omics data such as whole exome sequencing and RNA sequencing — two of the most widely adopted techniques in cancer research. The proposed pipelines focus on uncovering two primary categories of potential cancer targets (1): recurrent peptides presented by HLA class I molecules for cancer immunotherapies, and (2) synthetic lethality pairs, which could inform the development of targeted therapies, such as protein inhibitors (e.g., PARP inhibitors).

Herein, we show three different approaches to identify new targets for designing precision and personalized therapies for cancer patients, using our CRC patient cohort (NCT04994093) as an example (**Figure 1**) (23). We employ AI-based methods to identify patient-specific neoepitopes and synthetic lethality gene pairs.

## Methods

### Patient selection and sample collection

The patient cohort analyzed in this study was previously described in Bujak et al. (23). The cohort consisted of 100 CRC patients recruited from surgical oncology departments in Poland between October 2021 and December 2022. The original study protocol was approved by the local ethics committee (no. KB/430-87/21) and informed consent was obtained from all participants prior to enrollment in the study. The detailed criteria for the patient selection process were described previously (23). Briefly, the enrolled participants were ≥18 years of age, with a diagnosed and histopathologically confirmed advanced resectional CRC adenocarcinoma in active stage II, III, or IV. The biological samples obtained from the study participants included the FFPE block of the primary tumor tissue or histopathological slides derived from it, along with peripheral blood samples. The peripheral blood samples from each CRC patient were collected into PAXgene^®^ Blood DNA tubes (Qiagen, Hilden, Germany).

### Whole Exome Sequencing (WES) and RNA sequencing (RNAseq)

Nucleic acid extraction, WES, and total RNAseq were performed at CeGaT GmbH (Tuebingen, Germany). The FFPE block samples and peripheral blood samples were used for nucleic acid extraction. To maximize tumor content, DNA and RNA were isolated from FFPE after macrodissection of the tumor area, which was distinctly labeled by a pathomorphologist. For DNA/RNA isolation the AllPrep DNA/RNA FFPE Kit (Qiagen) or MagMax FFPE DNA/RNA Ultra (Thermo Fisher) were used. The AllPrep DNA/RNA FFPE Kit is optimized to reverse as many formaldehyde modifications as possible, without causing further degradation of DNA and RNA. For extracting DNA from blood, the QIA Symphony DSP DNA Mini Kit 96 (Qiagen) was used according to the manufacturer’s protocol.

The quality control and concentration of isolated genomic DNA (gDNA) and total RNA were assessed using the Qubit fluorometer with the Qubit dsDNA HS Assay Kit or RNA High Sensitivity (HS) Assay Kit (both from ThermoFisher Scientific, Waltham, MA, USA) according to the manufacturer’s protocol. For WES, 50 ng of gDNA from each sample was used to generate libraries with the Twist Human Core Exome Plus Kit (Twist Bioscience, San Francisco, CA, USA). For RNAseq, 10 ng of total RNA from each sample was used to generate libraries with the SMART-Seq Stranded Kit (Takara Bio, Kusatsu, Japan). The qualities of the generated libraries (both for WES and RNAseq) were verified using the 2100 Agilent Bioanalyzer with High Sensitivity DNA Kit and High Sensitivity RNA Kit (both from Agilent Technologies, Santa Clara, CA, USA). Next, the obtained libraries were paired-end sequenced using the NovaSeq 6000 platform (Illumina, San Diego, CA, USA) to achieve at least 150x and 300x (PBMC and FFPE samples, respectively) reading depth for >98% targeted bases.

Due to the poor quality of the RNA (DV200 < 30%), 29 samples were excluded from sequencing and further analyses. For 71 patients, full sequencing was performed, both for tumor tissue (WES & RNAseq for tumor) and for blood samples (WES normal). All of the aforementioned samples successfully passed the bioinformatic quality control analysis, which assessed library GC content, duplication levels, and base pair composition.

### Identification of neoepitopes with ARDentify and ARDisplay

In this study, a robust and comprehensive genomic analysis pipeline was employed to examine tumor and normal tissues. The initial processing of raw pair-end WES reads involved adapter trimming using Cutadapt (24), followed by alignment to the primary assembly genome (Ensembl GRCh38_95) using the Burrows-Wheeler Alignment (BWA) tool (25). To ensure data integrity, duplicate reads in the resulting BAM files were identified and removed using Picard processing tools (“Picard Toolkit.” 2019. Broad Institute, https://broadinstitute.github.io/picard/). Additionally, using the same toolkit, metadata was incorporated into the files by adding read groups. Quality refinement was further implemented through base quality score recalibration utilizing the Genome Analysis Toolkit (GATK) (26).

Somatic variant calling was carried out employing Strelka 2 (v2.9.7) (27), VarDict (v1.6.0) (28) and MuTect (included in GATK v4.3.0) (26), and only variants consistently reported by all three callers were selected for downstream analysis. Germline variant calling was performed using three independent variant callers: Strelka 2 (v2.9.7) (28), GATK (26) and Octopus (v0.7.4) (29). To ensure confidence in the variant calls included in the downstream analysis, we intersected the results consistently reported by all three callers using BCFtools (v1.10.2) (30). The variants for each sample were then annotated with ANNOVAR (v2018Apr16) (31) using RefSeq (32) and dbsnp150 (33) databases.

Furthermore, HLA class I typing was performed using Polysolver (34). First, all the variants that could generate TSAs were selected by filtering out synonymous mutations. Next, the processed WES and RNAseq data were used in a multifaceted approach adopted to assess various aspects of peptide-HLA (pHLA) interactions and select the most promising neoepitopes candidates. To enhance the selection process of neoepitopes with the highest chance of being recognized by the immune system and likely to elicit an immune response, we employed a systematic ranking strategy that considers, among other factors, the two crucial aspects of peptide presentation on the cell surface. Namely, using ARDisplay (35) we account for both the binding affinity between peptides and HLA molecules (using MHCflurry (36)) and for the peptide:HLA complex - the presentation on the cell surface. Notably, the ARDisplay model integrates patterns from a vast dataset of HLA ligandomics mass spectrometry experiments, enabling comprehensive characterization of the repertoire of peptides presented via HLA molecules. This holistic approach provides a deeper understanding of the entire antigen processing and presentation pathway, facilitating the identification of neoepitopes with superior immunogenic potential.

### CMS calculation

Consensus Molecular Subtypes (CMS) were assigned using the CMScaller tool as described elsewhere (37). Briefly, CMScaller is an R package that assigns CMS category to samples based on the expression profiles of cancer cells. It achieves this by using a filtered set of markers specifically enriched in certain cancer subtypes. CMScaller assigns a tumor to a specific category based on its gene expression profile. The confidence of this assignment is estimated by resampling the genes (1000 times) and checking for consistency. Samples that do not meet a certain confidence threshold are labeled as “not assigned”. The pipeline for processing RNA-seq data from tumor tissue to be used by CMScaller consisted of adapter removal using Cutadapt, followed by alignment to the Ensembl GRCh38_95 primary assembly genome with STAR (38). Quantification of gene expression levels was conducted using the RNA-Seq by Expectation-Maximization (RSEM) algorithm (39).

### Multimodal VAE for a comprehensive understanding of gene regulation with low-dimensional data representations

To develop a MVAE for description of genomic profiles of cancer patients, we utilized a comprehensive dataset comprising 1456 cell lines sourced from the Cancer Cell Line Encyclopedia (CCLE) (https://sites.broadinstitute.org/ccle/), complemented by gene essentiality/dependence information for 1095 of these cell lines obtained from the DepMap (https://depmap.org/portal/) database. Additionally, our study incorporated cancer patient data, drawing from a vast cohort of over 11,000 patients spanning 37 indications sourced from The Cancer Genome Atlas (TCGA) database (https://www.cancer.gov/tcga).

For the CRISPR genome-scale knockout screening scores on cell lines, we obtained the DepMap Public 23Q2 dataset, released in May 2023, containing the Chronos gene effect from the Achilles project (https://depmap.org/portal/achilles/) calculated for 19,000 genes. The TCGA data were acquired using the Bioconductor package TCGAbiolinks version 2.28.3, last updated on June 5th, 2023 (40). Gene expression quantification data were accessible for both cancer cell lines and patients, aligned to the hg38 reference genome, and provided in Transcripts per Million (TPM). We applied a logarithmic transformation on the TPM values using the formula log2(TPM+1), and exclusively considered protein-coding genes during processing. DNA methylation data were obtained as methylation beta value derived from Methylation Array technology utilizing Illumina 450K BeadChip microarray-based methylation platform.

To address the high dimensionality inherent in omics data, we adopted a strategy employing an encoder-based approach. Specifically, we developed separate VAEs (Variational AutoEncoders) tailored to each modality, here, limited to 1/gene expression, and 2/DNA methylation. This approach enabled us to extract important features while effectively and concurrently reducing the input dimensionality. To facilitate the integration of diverse omics data, we used a Multimodal VAE, a VAE model based on the concept of Product of Experts (41). Such a model enables the integration and enhancement of signals detected in individual components, as the two modalities influence each other, allowing for a more holistic and comprehensive understanding of gene regulation. The loss function is the Kullback-Leibler (KL) divergence of the combined experts (individual VAEs) plus the reconstruction losses of each modality. We reused the architecture and loss functions from the unimodal training scheme.

MVAE was implemented in Python 3.9 using the PyTorch v.2.0 library to perform fast operations on tensors and neural networks with GPU acceleration. Additionally, we have used the PyTorch Lightning v2.0.0 framework to streamline the training process of our models alongside the open-source MLflow platform to monitor metrics during model development and document progress. GPU-based computations were done on a machine equipped with NVIDIA Ampere A100 GPU cards with CUDA^®^ 8.6 architecture, 640 Tensor Cores, 6,912 CUDA^®^ Cores, and 40 GB HBM2 GPU Memory, and using cuDNN 8.5.0 and cudatoolkit 11.7.99. Standard Python libraries for data analysis and machine learning were used, inter alia, scikit-learn, pandas, numpy, matplotlib, and seaborn.

### Selection of recursive variants for synthetic lethality

Two distinct methodologies were employed to identify genetic variations potentially associated with synthetic lethality. For the precision medicine strategy, recurrent somatic mutations present in at least 3 out of 71 CRC patients were identified using the dplyr package (v. 1.1.3) in R. Subsequently, the presence of these mutations was assessed in the CCLE (42) database containing somatic variants, copy number variants, and CRISPR knockout survivability screens. Lastly, the presence of the selected pairs of mutations was verified in two TCGA cohorts (TCGA-COAD and TCGA-READ). As for the personalized SL approach, our MVAE model was utilized to extract features from gene expression data, facilitating the identification of cancer cell lines (CCLs) that exhibit similarities to the patient data. Although RNA-seq embeddings were prioritized due to their stronger predictive performance, methylation data played a key role during training, providing a more nuanced representation of gene regulation and accounting for epigenetic factors that influence expression patterns. The subsequent analysis involved the identification of all recurrent SNVs and copy number variations (CNVs) found within the corresponding CCLs. For each recurrent variant, a study group consisting of CCLs with the mutation and a control group of CCLs without the mutation were generated. The study group had to be composed of at least 7 CCLs to proceed to the subsequent steps of the analysis. Limma R package v.3.56.2 (43) was used to concurrently estimate the mean difference in the dependency score (a numeric value representing the essentiality of a gene in a CCL) for each gene between the study group and the control group. The p-values and p-adjusted values were computed using the empirical-Bayes moderated t-statistics. For each analysis, a volcano plot was generated with ggplot2 v.3.4.3.

## Results

### Cohort characterization

The cohort consisted of 71 patients diagnosed with colorectal cancer, with a mean age of 68 years (SD = 9). Of these, 65% were male and 35% were female (**Table 1**). Around half of the patients within the cohort were at stage II at the time of diagnosis while another half were at stage III and IV (**Table 1**). Utilizing CMScaller, we determined CMS of colorectal cancer for our cohort of patients (**Table 1**, **Supplementary Figure 1**) (9).

**Table 1:** Cohort description (n=71).

		Number of patients	% of total
Age	<=65	22	31.0
	>65	49	69.0
sex	M	46	64.8
	F	25	35.2
tumor location	rectal	21	29.6
	colon	29	40.8
	NA	21	29.6
stage	I*	0	0.0
	II	35	49.3
	III	20	28.2
	IV	16	22.5
CMS	CMS1	15	21.1
	CMS2	23	32.4
	CMS3	3	4.2
	CMS4	22	31.0
	NA	8	11.3
BRAF	MUT	6	8.5
	WT	65	91.5
KRAS	MUT	29	40.8
	WT	42	59.2

*Stage I was an exclusion criterion.

Among the cohort, 15 patients were classified under category CMS1 - hypermutated, microsatellite unstable with a strong immune activation; 23 patients fell into category CMS2 - epithelial, chromosomally unstable with a marked WNT and MYC signaling activation; 3 patients were identified in category CMS3 - epithelial, evident metabolic dysregulation; and 22 patients were categorized as CMS4 - mesenchymal, prominent transforming growth factor β activation, stromal invasion, and angiogenesis. For 8 patients the category was not assigned as their results showed mixed or intermediate features. The cohort was compared to 2 published datasets, containing 4 cohorts in total. In most cases, the new cohort followed previously observed trends (**Supplementary Figure 1**).

### Pathogenic germline variants

To verify if any of the patients harbored germline variants that were previously linked to CRC incidence, we checked the intersection between all germline variants from our patients and the database of 871 germline cancer variants (44). No variants directly linked to CRC were identified, indicating the spontaneous character of the disease. However, in 5 patients, we found 4 missense variants that were present in one or two of them and had been linked to increased cancer incidence. These variants were identified for the following genes: *ATM* R2832C (chr11:108345818 C>T), *SDHA* R31* (chr5:223509 C>T), *PMS2* R9* (chr7:6003982 G>A), *DKC1* S280R (chrX:154769233 A>C). Importantly, the occurrence of these mutations in our cohort was ~7%, which was consistent with the 8% found in the cohort of 10,389 patients from Huang et al. (44).

### Somatic landscape

The analysis of our cohort unveiled the most frequently mutated genes (**Supplementary Table 1**, **Table 2**), with *APC* being the most frequently mutated one (31 patients), followed by *KRAS*, *SYNE1* (29 patients, 41% each), and *TP53* (24 patients). Moreover, the top 10 most prevalently mutated genes in CRC (45), ie. *APC*, *TP53*, *KRAS*, *PIK3CA*, *FAT4*, *FBXW7*, *CSMD3*, *BRAF*, *LRP1B* and *SMAD4*, were all recurrently identified in our cohort (45) (**Table 2**).

**Table 2:** Comparison of the prevalence of mutated variants in our cohort and (45).

	Percentages of mutated variants	
Gene	CRC Literature	CRC Ardigen cohort
*APC*	66%	44%
*TP53*	52%	34%
*KRAS*	43%	41%
*PIK3CA*	30%	21%
*FAT4*	19%	27%
*FBXW7*	16%	14%
*CSMD3*	15%	30%
*BRAF*	14%	8%
*LRP1B*	12%	16%
*SMAD4*	11%	10%

Next, using the consensus-based approach to variant calling (section Identification of neoepitopes with ARDentify and ARDisplay), we identified 32 recurrent mutations present in at least 3 patients (**Supplementary Table 2**). Notably, the most prevalent variants were detected in *NBPF1* A955V (chr1 g.16565782G>A, 14 patients), *KRAS* G12D (chr12:25245350 C>T, 8 patients) and *BMPR2* N583Kfs*6 (NM_001204.7, 7 patients). To identify potential neoepitopes as therapy targets, we assessed whether any of the detected somatic variants could be presented on the tumor cell surface within the patient’s HLA class I. Leveraging our in-house presentation model, ARDisplay, we identified 8775 unique neoepitopes with a high probability of being presented. Out of them, 89 were shared by at least two patients, while the vast majority were present only in individual patients (**Supplementary Table 3**). Even the most prevalent variants did not yield commonly shared neoepitopes due to the significant variability in HLA class I alleles expressed by patients carrying these mutations. This heterogeneity in HLA expression across individuals limited the generation of universally recognized neoepitopes, making it challenging to identify common immunogenic targets among the cohort.

### Cohort gene essentiality

**Figure 2:**
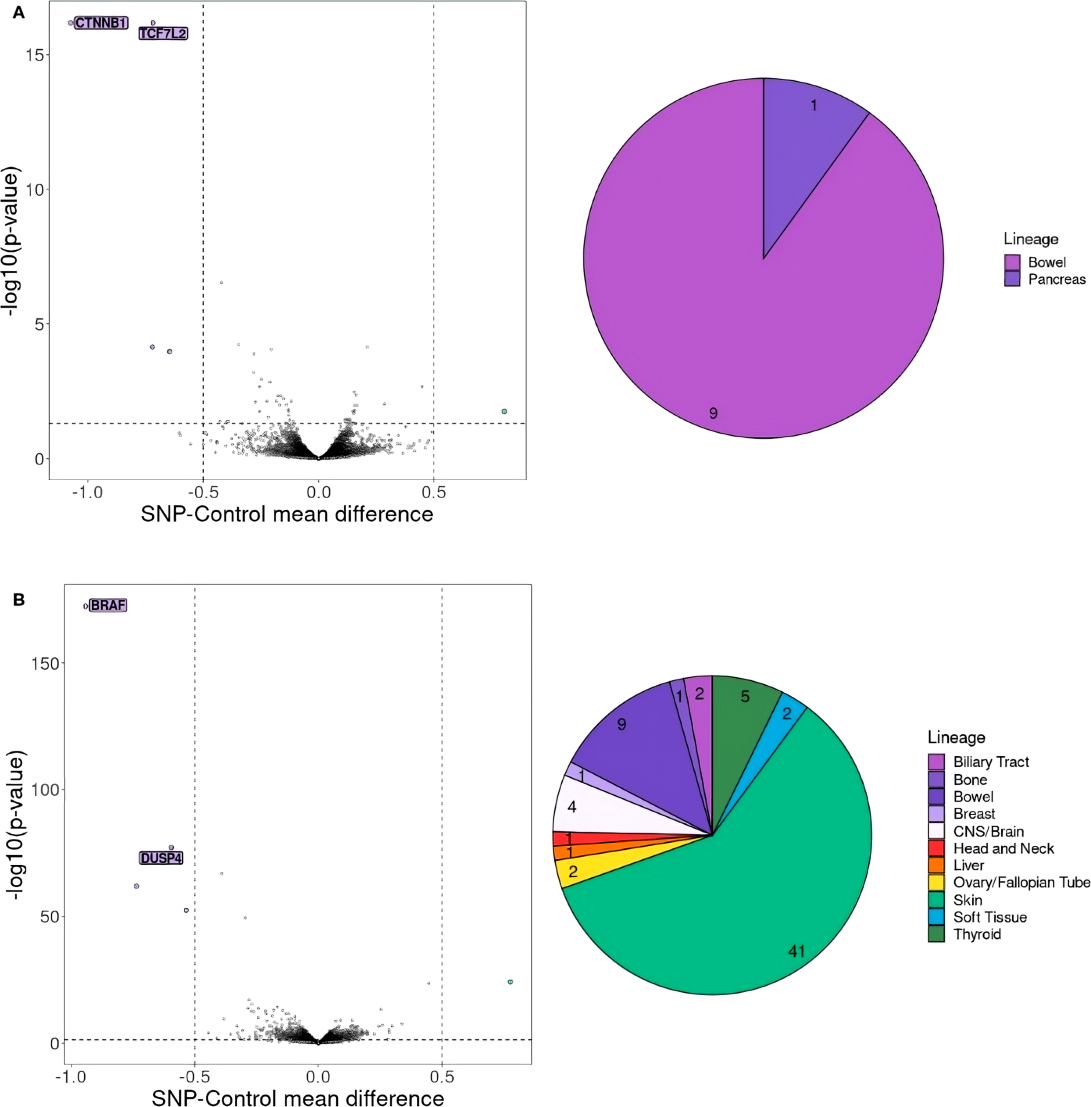
Gene essentiality for cell lines with **(A)** *APC* T1396Nfs*3 mutation, **(B)** *BRAF* V600E mutation. On the left selected essential genes for cell lines with **(A)** *APC* T1396Nfs*3 mutation (chr5:112840254 G>GA) and **(B)** *BRAF* V600E mutation (chr7:140753336 A>T) are shown. The genes that should be considered crucial for cell proliferation are the ones with a mean difference of dependency score below -0.5 or above 0.5. A score below -0.5 indicates genes whose absence negatively affects cell proliferation, whereas a score above 0.5 indicates genes whose knock-out positively impacts cell proliferation. A pie chart on the right shows the number and proportion of CCL lineages with **(A)** *APC* T1396Nfs*3 and **(B)** *BRAF* V600E mutation.

To explore potential targets for cancer therapies beyond HLA-dependent mechanisms, we undertook an analysis of gene essentiality within our cohort. From the 32 recurrent somatic variants identified in at least 3 patients, we selected the most common ones and analyzed further 5 of them which were detected in at least 7 cell lines from CCLE. These variants included *PIK3CA* E545K (chr3:179218303 G>A), *APC* T1396Nfs*3 (chr5:112840254 G>GA), *BRAF* V600E (chr7:140753336 A>T), *KRAS* G12A (chr12:25245350 C>G) and *KRAS* G12D (chr12:25245350 C>T). Interestingly, for mutations in *APC* and *BRAF*, we identified essential genes previously described in the literature as promising targets for cancer therapy. Specifically, for *APC* T1396Nfs*3 mutation, two essential genes *CTNNB1* and *TCF7L2* were discovered (**Figure 2A**) (46). Similarly, for *BRAF* V600E, the essential gene *DUSP4* was found (**Figure 2B**) (47). However, for the 3 remaining variants, no such genes were identified. In an analysis of TCGA colon and rectum adenocarcinoma samples (n=196), the *APC* variant T1396Nfs*3 was detected in 3 cases. None of these samples exhibited variants in *CTNNB1* or *TCF7L2*. Additionally, the *BRAF* V600E mutation was found in 26 samples. In one of the patients we also found a co-occurring *DUSP4* variant (chr8:29338496 A>AC) which was however heterozygous and its full functional impact may be limited.

### Personalized gene essentiality with MVAE

**Figure 3:**
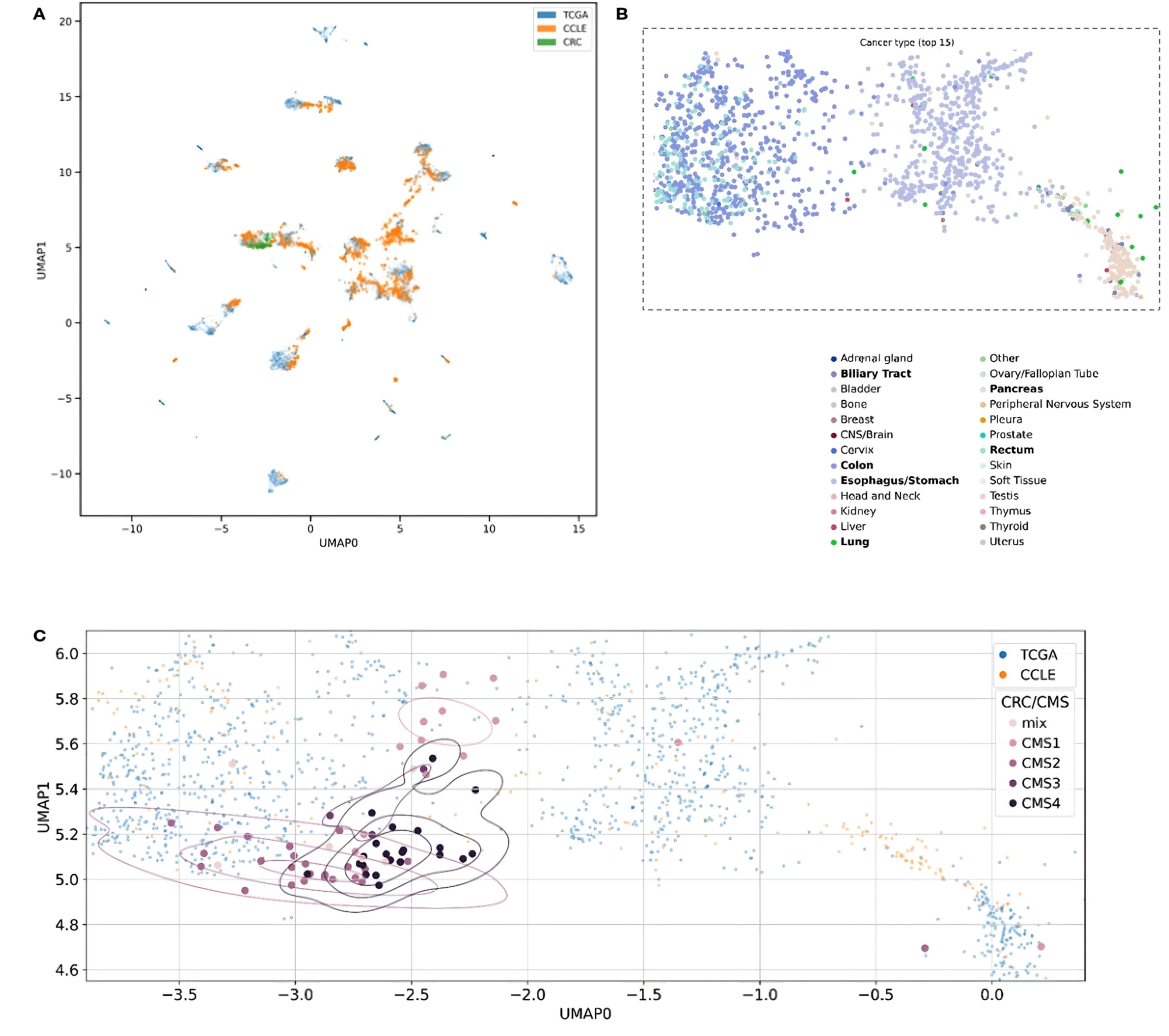
Two-dimensional UMAP visualization derived from the MVAE model’s representation of multi-omics data. Each point in the plots corresponds to either a single cell or a patient sample. The scatter plot allows to observe patterns and relationships between different samples. **(A)** Data points are color-coded to distinguish between different data sources, such as cancer cell lines (CCLE) and patient samples (TCGA and CRC). The overlapping colors reflect the creation of a unified representation, aided by MVAE model predictions, and the removal of batch effects. **(B)** Color encoding is based on cancer types, with the 15 most frequent types selected for better legibility. Additionally, we restricted the embedding space to areas with all CRC patients and zoomed in. Legend labels marked in bold are cancer types with at least 10 data points in the selected area. **(C)** Each point in the plots corresponds to either a single cell (CCLE) or a patient sample (TCGA and CRC). Data points corresponding to CRC patients from our cohort are color-coded according to their Consensus Molecular Subtype (CMS) classification.

Finally, we assessed the potential utility of our Multimodal VAE model in a more personalized approach by the identification of possible targets tailored to specific patients. Initially, we mapped RNAseq expression results for each patient to CCLs, observing that all patients clustered with cell lines derived from colon or rectal cancer (**Figures 3A, B**). These Figures represent the UMAP (Uniform Manifold Approximation and Projection) space, ie. a dimensionality reduction technique that enables to effectively visualize 32-dimensional data in a 2-dimensional space.

Notably, the overlapping colors in **Figure 3A** demonstrate the creation of a unified representation, where cell lines and patient samples blend together without forming separate clusters. This suggests that the representation is organized by cancer type and genomic characteristics rather than by the origin of the samples. This cohesive representation is achieved through the MVAE model with the successful removal of batch effects among samples of diverse origin. This approach provides valuable insights into the underlying biological processes, in particular, by enabling the alignment between *in vitro* models and tumor samples. Utilizing color encoding, based on cancer types (**Figure 3B**, the 15 most frequent types selected for better legibility), reveals that the structure of the clusters can be attributed to cancer type or tissue origin, despite these factors not being directly introduced to the model. This capability of the model to discern such differences enables the exploration of cellular heterogeneity.

Additionally, data points representing CRC patients from our cohort were color-coded based on the CMS categorization (**Figure 3C**). In the visualization, distinct patterns emerge in the distribution of CMS categories across the UMAP space. Notably, CMS4 samples appear clustered together, while the remaining CMS categories exhibit their own clusters. This observation underscores the significance of the gene signature and highlights the substantial heterogeneity of CRC, as revealed by genomic and transcriptomic profiling techniques.

**Figure 4:**
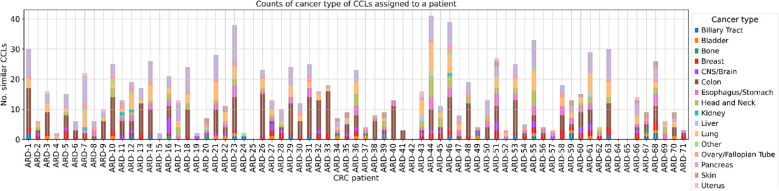
The number of CCLs classified as similar to each patient with cancer lineage discrimination. The stacked bar plot illustrates the number of cancer cell lines (CCLs) similar to each CRC patient with an additional distinction between the 15 most frequent cancer types. Similarity was determined based on the Euclidean distance in the 32-dimensional space obtained via the MVAE model, with a threshold set at the 1st percentile. Each bar represents a CRC patient (x-axis), and the height of the bar indicates the number of similar CCLs (y-axis). Segments in each bar are color-coded according to the cancer type of cancer cell lines they represent. The absence of bars on the plot for patients ARD-25 and ARD-42 is attributed to the lack of closely related CCLs, while for ARD-64 and ARD-65, predictions from the MVAE model were unavailable.

Subsequently, we determined the CCLs that mimic the biology of each patient (**Figure 4**, **Supplementary Table 4**). The plot reveals significant variation in terms of the number of CCLs that are the closest to the respective patients. Some patients, such as ARD-23, ARD-44, ARD-46, or ARD-55, exhibit substantial representation, with more than 30 similar cell lines each. Conversely, patients ARD-4, ARD-15, ARD-19 and ARD-24 demonstrate comparatively limited representation.

**Figure 5:**
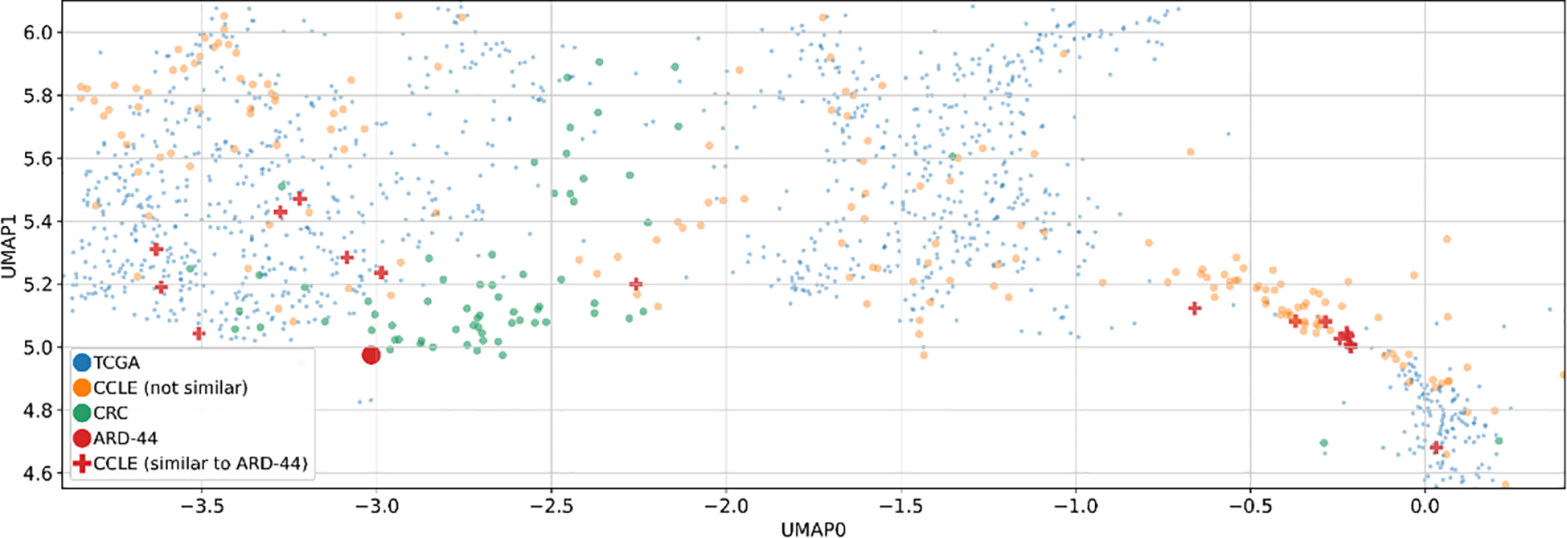
Two-dimensional UMAP visualization derived from the MVAE model’s representation of multi-omics data. Two-dimensional UMAP visualizations derived from MVAE representations of multi-omics data. Each point represents either a single cell line (CCLE) or a patient sample (TCGA and CRC). Additionally, similarities between a specific CRC patient (ARD-44) and various cancer cell lines (CCLs) are depicted on the plot, based on the Euclidean distances in a 32-dimensional space obtained via the MVAE model. This focused visualization provides insights into the similarities between CRC patients and CCLs, particularly in the context of colon cancer.

For further analysis, we focused on patient ARD-44 due to the highest number of similar CCLs (>40, **Figure 4**). Specific cell lines similar to this patient are depicted on the plot (**Figure 5**, **Supplementary Figure 2**), although they may not always be the closest points on the plot. This suggests that the relationship between the patient and CCLs may not always be directly observable in the scatter plot. The underlying 32-dimensional space harbors a wealth of additional biological insights beyond what can be visualized in 2D. While the scatter plot provides a simplified view of the similarities between CRC patients and CCLs, it is the MVAE embedding that encapsulates an understanding of the biological landscape.

**Figure 6:**
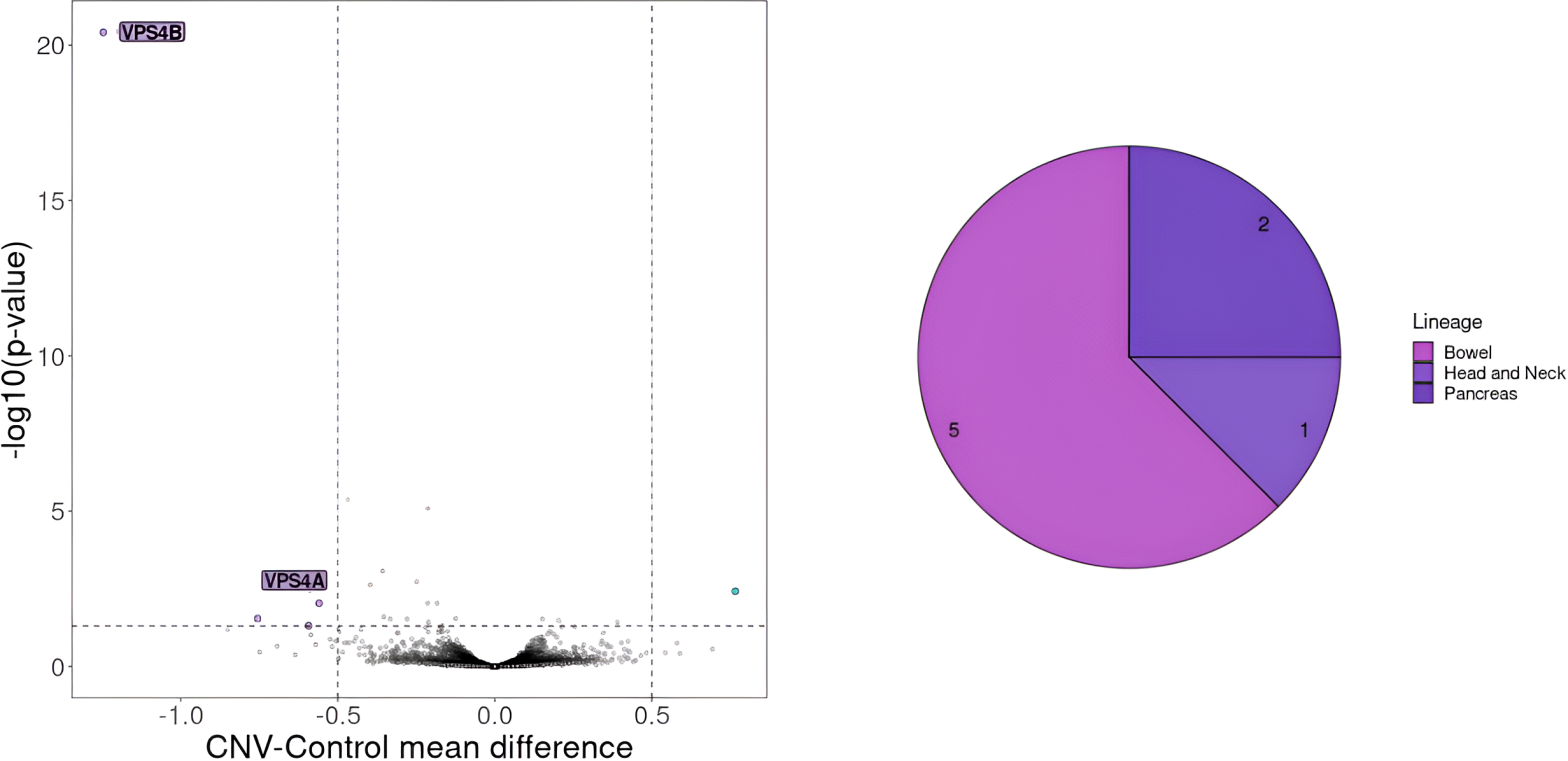
Gene essentiality for cell lines with CNV on chromosome 18 encompassing *VPS4B* gene. On the left selected essential genes for cell lines with deletion of more than 100 genes on chromosome 18 including VPS4B a gene known for its synthetic lethality effect in collaboration with VPS4A. The genes that should be considered crucial for cell proliferation are the ones with a mean difference of dependency score below -0.5 or above 0.5. A score below -0.5 indicates genes whose absence negatively affects cell proliferation, whereas a score above 0.5 indicates genes whose knock-out positively impacts cell proliferation. On the right CCLs lineages carrying the deletion.

After identifying a set of cell lines designated as similar to the patient, we examined all recurrent SNV and CNV present in more than 20% of these cell lines. This analysis revealed 16 SNVs and 16 recurrent large deletions occurring in at least 5 CCLs (**Supplementary Tables 5**, **6**). Further investigation using volcano plots highlighted *VPS4B* and *VPS4A* as potential targets associated with reduced cell proliferation (**Figure 6**). Notably, both genes constitute a synthetic lethality pair, as previously described (48).

Subsequently, we determined genes crucial for cell survival and examined whether any of the recurrent SNVs or CNVs observed in the CCLs were also present in the patient. We then explored whether patient ARD-44 harbored a deletion in either *VPS4A* or *VPS4B*. By analyzing the frequency of heterozygous mutation (15 variants) in *VPS4B* and its neighboring genes in tumor versus normal tissues, we concluded that the patient’s tumor exhibited a mosaic deletion of *VPS4B* (**Supplementary Figure 3**).

## Discussion

CRC remains one of the most prevalent cancer types and its incidence continues to rise globally (4). Although CRC survival rates are increasing for the early stages, most of the available treatments prove insufficient in the metastatic stage, especially for patients that are not classified to CMS1 with MSI and high TMB (~80% of patients in our cohort) (6, 7, 49, 50). All this makes the development of innovative personalized approaches and precision therapeutics against CRC an important research subject, that in the future might save patients’ lives. In this publication, we introduced various pipelines created by Ardigen aiming at the identification of targets for novel CRC therapies.

The study was performed on a group of 71 patients of Polish nationality with confirmed advanced resectional CRC adenocarcinoma in active stages II, III or IV. In comparison to the other cancer types, the genetic landscape of CRC in the Polish population has not been extensively studied yet (51). Based on multiple types of NGS data, this study analyzed different characteristics of CRC (e.g. germline mutations, somatic mutations, CMS). Our analyses revealed that CMS2 and CMS4 are the most prevalent subtypes in our CRC cohort, while CMS1 and CMS3 are less frequent, which is consistent with the literature (9) (52), (**Supplementary Figure 1**). Furthermore, when we compared the landscape of somatic and germline mutations with other previously published cohorts, we concluded that our patient group did not differ significantly from other non-Polish populations (**Table 2**) (9, 44, 45, 52).

Subsequently, we verified whether there are shared neoepitopes that could be promising targets, through the use of ARDentify, Ardigen’s proprietary platform. This platform facilitates the identification of neoepitopes from tumor and blood WES data and prioritizes them using another proprietary presentation model called ARDisplay. ARDisplay has been trained on a comprehensive set of curated mass spectrometry data (53). As previously shown by the Rosenberg team, neoepitopes are rarely shared between CRC patients (54) and the analysis of the Polish cohort revealed a correlation with these results, as the detected neoepitopes were shared by a maximum of 2 patients. Out of 8 patients with KRAS G12D mutation, two had HLA-A03:01 or HLA-A11:01 type and could be potential candidates for TCR-Ts therapy that is being developed (55). Additionally, all 8 patients with KRAS G12D mutations could participate in clinical trials and potentially benefit from specific inhibitor therapies targeted against this mutation or pan-KRAS mutation vaccines (56).

Nevertheless, in most CRC patients, it would be more optimal to focus on more personalized approaches, e.g. Tumor Infiltrating Lymphocytes transfer therapy (57) or neoepitope vaccines (58), as no recurrent neoepitopes are present. This can be further supported by the notion that CRC is one of the cancer types with the highest tumor mutational burden (TMB), which increases the probability of finding neoantigen-specific T cells that can be further expanded *in vitro* and then delivered to the patient (59, 60). This is especially true for CMS1 patients, where in our cohort the mean number of neoepitopes equals 347 vs CMS2 - 4 with a mean of 60 neoepitopes per patient.

Next, using our in-house approach we checked if any synthetic lethality pairs could be used for a substantial number of patients in our cohort. Interestingly, the proliferation of cell lines with *APC* T1396Nfs*3 mutation was heavily dependent on *CTNNB1* and *TCF7L2* genes, while *BRAF* V600E was dependent on *DUSP4*, respectively. The pairs *APC-CTNNB1* and *BRAF-DUSP4* were previously described (47) (61–64), validating our approach to SL gene pairs identification. Additionally to the well-known SL pairs, we detected the *APC-TCF7L2* pair. This pair has been recently described as a new potential candidate for SL. However, it has not been confirmed experimentally in any *in vitro* test so far (46). *APC* and *TCF7L2* are part of the *Wnt/β-catenin* pathway. The *APC* gene encodes a critical tumor suppressor protein that functions as a negative regulator of the *Wnt* signaling pathway and it exerts its inhibitory effect on *β-catenin* (65). The *TCF7L2* gene encodes a High Mobility Group (HMG) box transcription factor critical for the Wnt signaling pathway. While implicated in blood glucose regulation with variants linked to type 2 diabetes risk, TCF7L2’s primary function lies within the intestine (66). In healthy intestinal epithelium, TCF7L2 acts as the sole mediator of canonical Wnt/β-catenin signals in stem and progenitor cells, driving their proliferation and maintaining tissue homeostasis (67, 68). This essential role positions TCF7L2 as a potential tumor promoter (69). However, TCF7L2 presents a paradoxical role in CRC. Despite its growth-promoting function in healthy tissue, TCF7L2 is frequently mutated in CRC (~10%) (70). However, it is not specified whether inactivating mutations in *TCF7L2* and *APC* can coexist, similarly to the *APC* and *CTNNB1* pair. In instances of *CTNNB1* mutation, inactivating *APC* mutations are never present (62). We believe that *APC-TCF7L2* should be further studied as a novel putative SL pair.

We tested our model’s ability to align cancer samples to the corresponding CCLEs by cluster colocalization of TCGA samples and DepMap cell lines (**Supplementary Figure 5**). Additionally, we performed an external validation by utilizing Multimodal VAE on one case to see if this particular patient could benefit from a personalized approach to cancer therapy. This framework enabled us to construct a robust joined representation of the input omics data, accommodating scenarios where certain modalities were missing or incomplete. For example, in the case of CRC patients, where DNA methylation data was absent, the MVAE model provided a mechanism for making predictions disregarding the missing information or even imputing it based on the remaining modalities. Using this model we were able to select 41 CCLs that can represent the cancer type of the selected patient (ARD-44). In 8 cell lines, we identified a deletion on chromosome 18 encompassing the *VPS4B* gene that was also present in our patient. Additionally, *VPS4A* was found by our analysis as a potential SL pair for *VPS4B*, which is consistent with the available literature (48). All this validates our MVAE model and implicates its usefulness in finding novel SL pairs.

Importantly, the heterogeneous number of similar CCLs found for different CRC patients by our MVAE model emphasizes the necessity for diverse and personalized approaches to better understand and treat the disease. Patients with limited representation may showcase disease subtypes or molecular profiles that are not as evident in the existing cancer cell lines. As expected, colon CCLs represent a substantial part of the similar CCLs for most of the patients (**Figure 5**). Additionally, the presence of CCLs derived from lung, pancreas, and other cancer types among similar CCLs may suggest that some CRC tumors might be sensitive to therapeutics used in cancer types derived from other tissues. While the study of common molecular features and shared pathways across cancer types may lead to the discovery of novel therapeutic strategies applicable to CRC and other cancers, the study of individual molecular patterns can help plan a highly personalized treatment plan and predict response to therapy. The individual approach, in particular, can facilitate clinical trials by using the MVAE model to stratify patients and predict potential adverse effects, thereby enhancing patient safety and increasing the benefit of participation.

In the future, additional applications of the MVAE model such as e.g. expansion into animal models with an attempt to map them to CCLs and cancer patients or integration of multi-omics data from other diseases, e.g. Parkinson’s disease might render our model useful in the identification of novel targets for diseases outside of cancer. Lastly, it is worth noting that the addition of different representations of multi-omics data like copy number variants and single nucleotide variants might further ameliorate MVAE models’ performance.

Herein, we presented 3 different approaches to the identification of possible targets for cancer patients using our in-house models: ARDentify, ARDisplay and an MVAE-based model. Notably, in every approach we have gained valuable insights into our cohort of 71 CRC patients, e.g. 1/*APC-TCF7L2* synthetic lethality pair that could be used in some carriers of variants in *APC* from our cohort, 2/*VPS4A-VPA4B - a* known synthetic lethality pair that was identified as a potentially personalized approach to one of our patients, importantly this gene pair could be an interesting target not only for CRC patients but also for carriers of the same CNV in other tumors and 3/a plethora of neoepitopes for every case that could be useful in personalized immunotherapies. Importantly in analysis 2, our findings indicate that the use of advanced techniques, such as MVAE and batch correction, results in a more comprehensive and versatile approach in comparison with other existing methods like Celligner (17). Since a direct cross-validation of the unsupervised model is not feasible on a dataset constructed using our approach, we use additional meta-data on cancer type as an alternative means of validating the model’s performance and generalizability as shown by the clustering of the cell lines in our model **Supplementary Figure 5**). The enhanced performance can be attributed to the joint representation of multi-omics data (RNAseq and methylation data), addressing the knowledge gaps identified in previous studies. It is worth noting that the authors of Celligner also indicate that the use of more explainable models based on VAE is a better approach than what they implemented in Celligner v1. This insight has led them to start incorporating such models in Celligner 2.0. Our study aligns with this perspective and improves upon it, as we have integrated VAE-based methodologies to enhance explainability and performance. By leveraging these advanced, explainable models, we provide a more transparent and interpretable framework that addresses both the limitations of Celligner v1 and the evolving needs highlighted by recent advancements.

## Data Availability

The datasets presented in this article are not readily available because they contain proprietary information that is currently under further analysis and subject to confidentiality agreements. Requests to access the datasets should be directed to the corresponding author Anna Sanecka-Duin.
