# Supplementary material for: Harnessing the power of AI in precision medicine: NGS-based therapeutic insights for colorectal cancer cohort
Source: Front Oncol. 2024 Oct 7;14:1407465. doi: 10.3389/fonc.2024.1407465 (PMC11491396; doi:10.3389/fonc.2024.1407465)
Supplement: Supplementary file 1 [file DataSheet1.pdf]

Supplementary Material

Harnessing the Power of AI in Precision Medicine: NGS-Based Therapeutic Insights for Colorectal Cancer Cohort

Victor Murcia Pienkowski, Piotr Skoczylas, Agata Zaremba, Stanisław Kłęk, Martyna Balawejder, Paweł Biernat, Weronika Czarnocka, Oskar Gniewek, Łukasz Grochowalski, Małgorzata Kamuda, Bartłomiej Król-Józaga, Joanna Marczyńska-Grzelak, Giovanni Mazzocco, Rafał Szatanek, Jakub Widawski, Joanna Welanyk, Zofia Orzeszko, Mirosław Szura, Grzegorz Torbicz, Maciej Borys, Łukasz Wohadlo, Marta Ewa Słomka, Michał Wysocki, Honorata Stadnik, Marek Karczewski, Beata Markowska, Tomasz Kucharczyk, Marek J. Piatek, Maciej Jasiński, Michał Warchoł, Jan Kaczmarczyk, Agnieszka Blum, Anna Sanecka-Duin*

*** Correspondence:** Corresponding Author: anna.sanecka-duin@ardigen.com

1 Supplementary Data

2 Supplementary Figures and Tables

2.1 Supplementary Figures

Figure S1

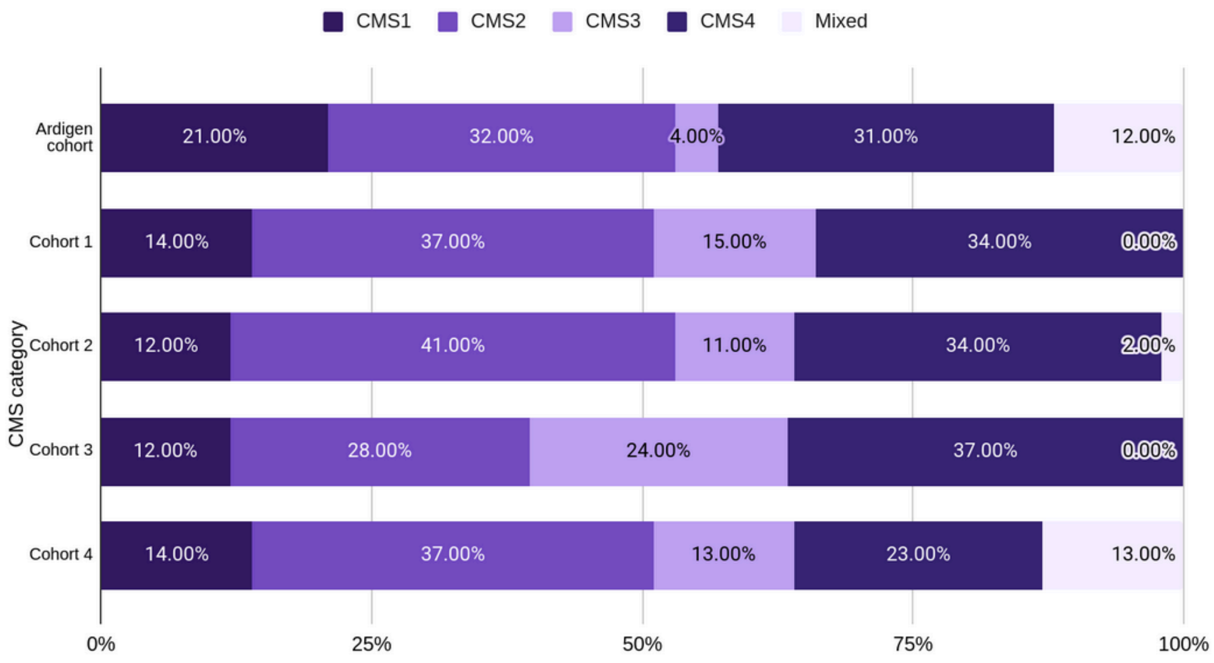

Supplementary Figure 1. The comparison of Ardigen cohort with previously published cohorts based on CMS frequency. The Ardigen cohort follows the expected trends in CMS category frequency. The analyzed cohort was compared to 4 other publicly available sets: Cohort 1 - total of 438 patients by Stintzing et. al. (1); Cohort 2 - total of 315 RAS wild-type tumor samples; Cohort 3 - total of 123 RAS mutated tumor samples and Cohort 4 - cohort published by Guinney et al. (2).

Figure S2

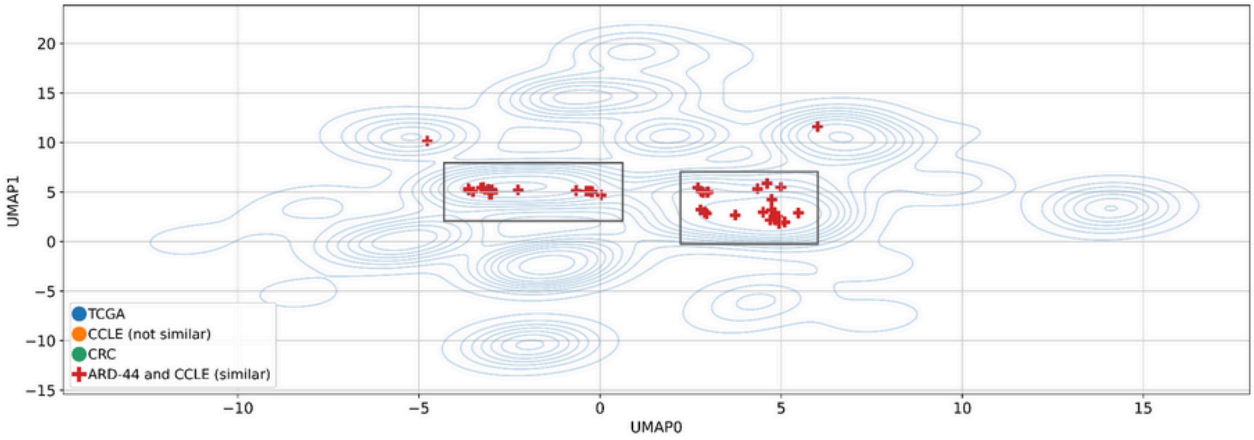

Supplementary Figure 2. Two-dimensional UMAP visualization derived from the MVAE model's representation of multi-omics data. Zoom out of Figure 4. Each "+" marker represents CCLs similar to the CRC patient (ARD-44), based on the Euclidean distances in a 32-dimensional space obtained via the MVAE model. Two distinct clusters are visible. The cluster on the left consists of CCLs from rectum and colon, whereas the cluster on the right encompasses CCLs derived from other tissues.

Figure S3A

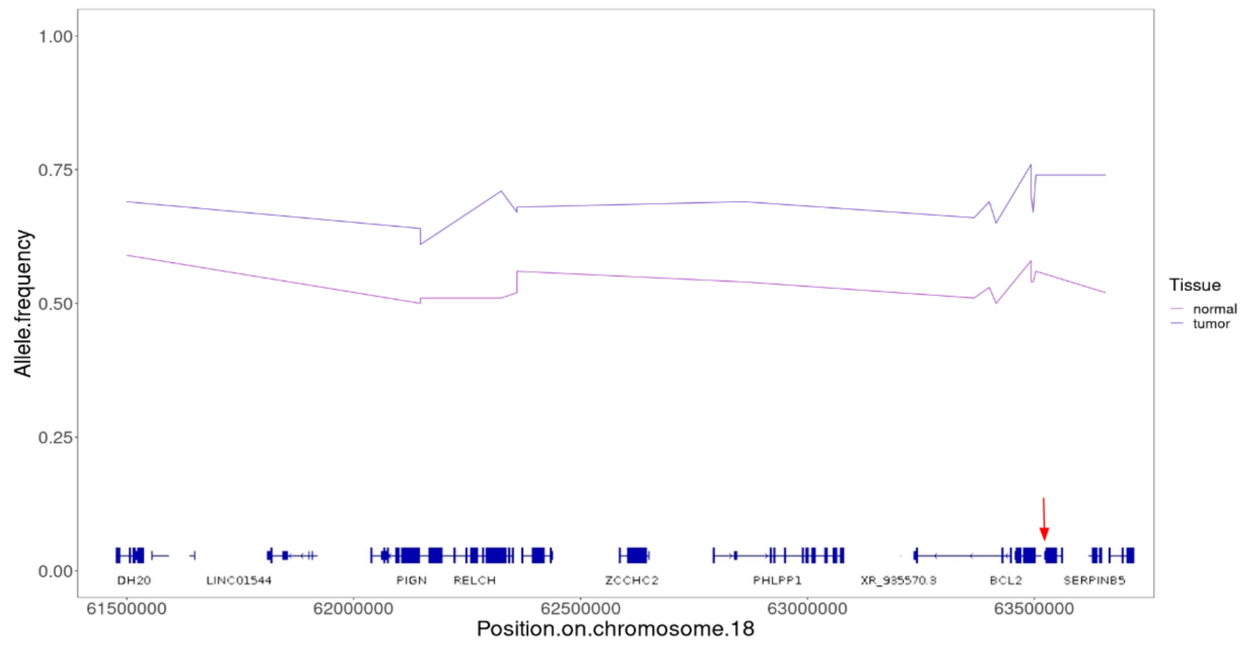

Figure S3B

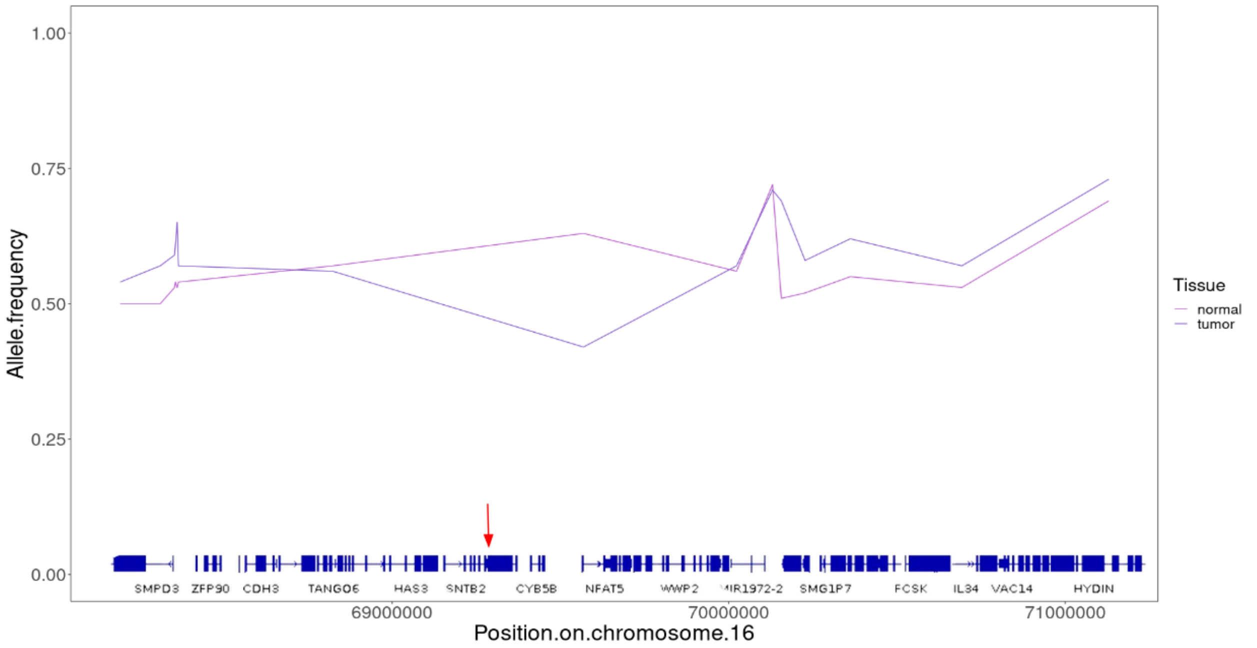

Supplementary Figure 3. Comparison of Allele frequency of heterozygous variants for normal and tumor samples from patient ARD-44. Allele frequency of heterozygous variants present in both normal and tumor tissue on chromosome (A) 18:61499308-63656799 and (B) 16:68191612-71129676. The red arrow indicates the position of gene (A) VPS4B and (B) VPS4A. Visibly, the frequency differs between the tumor and normal tissue in (A) suggesting a deletion in this region, while in (B) the frequency is similar between the tumor and normal tissue excluding the NFAT5 gene.

Figure S4

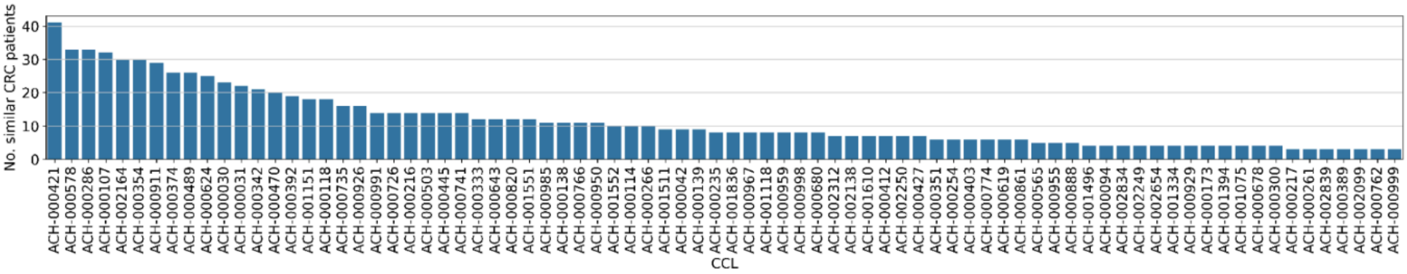

Supplementary Figure 4. The number of CRC patients that are similar to the corresponding cancer cell line. The bar plot represents the number of CRC patients similar to the cancer cell line (CCLE, top 80). The similarity is based on the Euclidean distance in the 32-dimensional space obtained via the MVAE model, with a threshold set at the 1st percentile. Each bar represents a cancer cell line (x-axis), ordered by the count of similar CRC patients (y-axis), facilitating the identification of cell lines with high numbers of similar patients. This ordering suggests potential candidates for further investigation or experimental use, as they exhibit stronger associations with CRC patients compared to others. The visualization aids in prioritizing cell lines for further studies and highlights their relevance in understanding CRC pathogenesis.

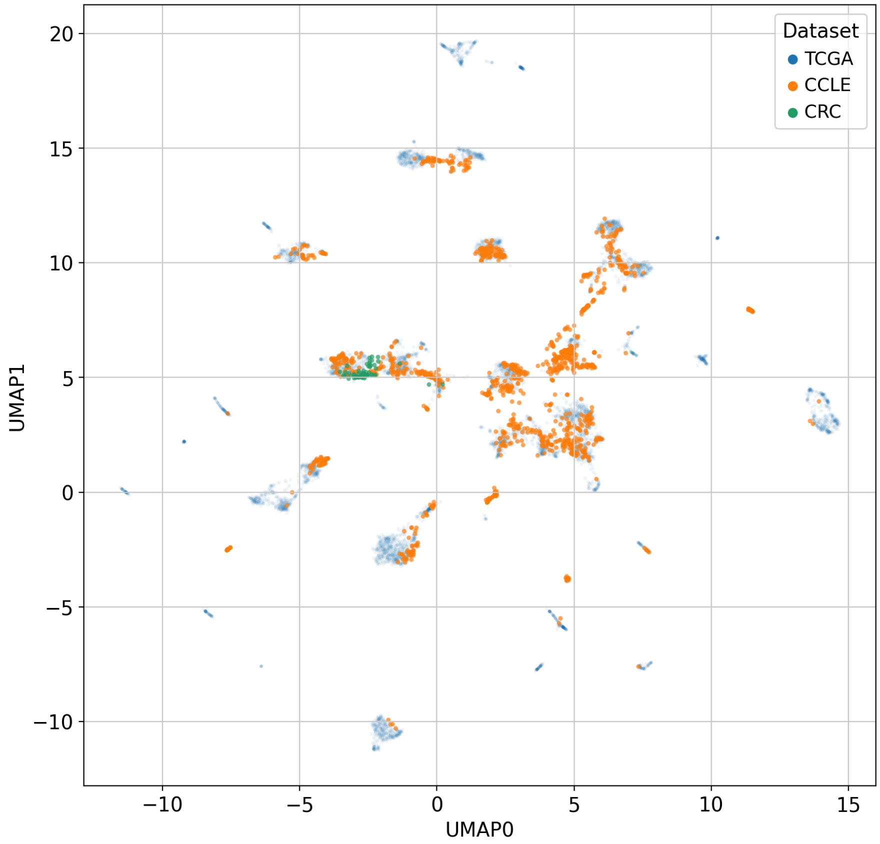

Supplementary Figure 5. UMAP 2D projection of multi-omics data representations obtained with Multimodal Variational Auto Encoders. The model identifies various clusters among cells, among others, CRC patients are clustered in the region where cell lines derived from colon and rectal cancer are present. Moreover, a general alignment of TCGA data and CCLE suggests the functionality of the MVAE model that generates clusters representing different cancer types even though this information was not provided into the model.

2.2 Supplementary Tables (Submitted as a separate excel files)

Supplementary Table 1. The list of the most frequently mutated genes.

Supplementary Table 2. Recurrent mutations present in at least 3 patients.

Supplementary Table 3. The list of neoepitopes shared by at least two patients

Supplementary Table 4. List of CCLs representative for each patient

Supplementary Table 5. The list of cell lines with and without deletion for 16 recurrent variants

Supplementary Table 6. The list of cell lines with and without deletion for 16 recurrent deletions

References

1. Stintzing S, Wirapati P, Lenz HJ, Neureiter D, Fischer von Weikersthal L, Decker T, et al. Consensus molecular subgroups (CMS) of colorectal cancer (CRC) and first-line efficacy of FOLFIRI plus cetuximab or bevacizumab in the FIRE3 (AIO KRK-0306) trial. Ann Oncol. 2019 Nov 1;30(11):1796-1803.
2. Guinney J, Dienstmann R, Wang X, de Reyniès A, Schlicker A, Soneson C, et al. The consensus molecular subtypes of colorectal cancer. Nat Med. 2015 Nov;21(11):1350-6.
